# First person – Igor Limaev

**DOI:** 10.1242/bio.062870

**Published:** 2026-09-10

## Abstract

First Person is a series of interviews with the first authors of a selection of papers published in Biology Open, helping researchers promote themselves alongside their papers. Igor Limaev is first author on ‘Transcriptional and histopathological profiling of skeletal muscle in Bla/J mice at the stage of dysferlinopathy manifestation’, published in BiO. Igor conducted the research described in this article while an undergraduate researcher in Olga N. Chernova's lab at Institute of Fundamental Medicine and Biology, Kazan Federal University, Kazan, Russia. Igor is now a research pathologist in the lab of Roman V. Deev at Avtsyn Institute of Human Morphology of the Petrovsky National Research Centre of Surgery, Moscow, Russia, investigating how rare muscle diseases work.

**Figure:**
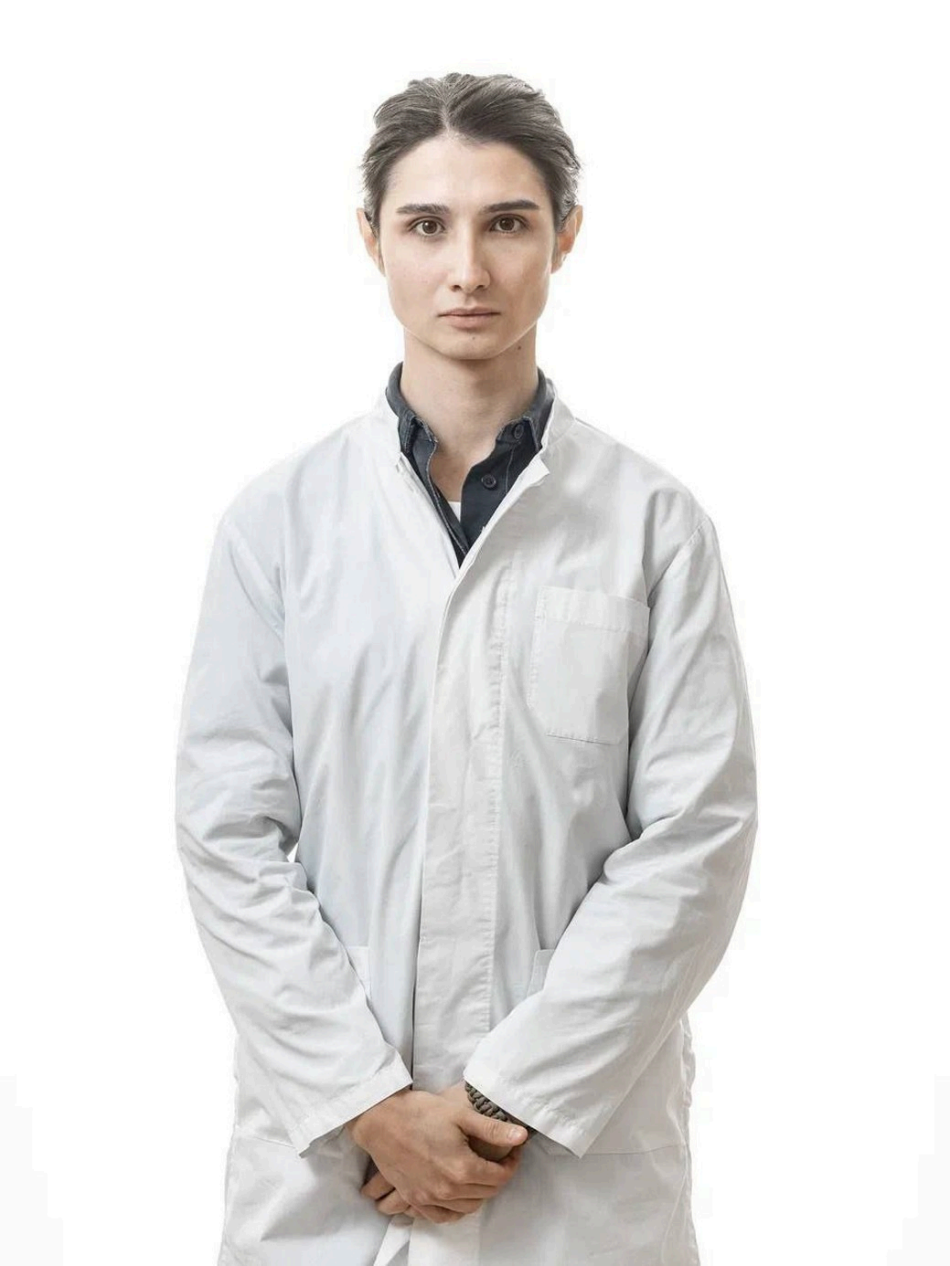
Igor Limaev

**Describe your scientific journey and your current research focus**

Ever since I was a child, I've had a deep drive to organise everything I learn about the world into a single, logical system. That is why I became a pathologist. I truly believe pathology is the fundamental trunk from which all other medical sciences grow. I specifically chose the niche field of muscular dystrophies because it is mysterious and largely uncharted territory. In an unexplored field like this, routine clinical diagnostics are never enough; you constantly have to invent new, non-standard scientific approaches to solve everyday clinical puzzles. Currently, I am applying this mindset to study a classic mouse model of dysferlinopathy. Although this model has been around for a long time, applying modern, high-resolution techniques allows us to discover something new every time. Ultimately, we need to completely understand if this model truly mirrors the human disease, which is absolutely critical before we use it to test new therapies for patients.

**Who or what inspired you to become a scientist?**

My primary inspiration is the fundamental desire to figure out how things work at their absolute core. I was very fortunate to have teachers who supported this curiosity and taught me to never settle for superficial answers. As a physician and researcher, I am also deeply dedicated to the study of orphan diseases – conditions that often go unnoticed simply because they do not affect the many. Yet for a doctor, the value of a human life is never measured in numbers. Solving these rare, intricate conditions is my way of contributing to a bigger picture.

**How would you explain the main finding of your paper?**

When muscle tissue wastes away, you usually expect to see two things at once: the physical damage under the microscope, and the specific ‘muscle-wasting’ genes actively turning on to drive that process. In our study of a rare muscle disease in mice, we could clearly see the muscles physically dying. Naturally, we expected the genes controlling the standard ‘cleanup’ system to be working in overdrive. Surprisingly, at the genetic level, they were quiet. The physical damage didn't match the genetic signals. Instead, we saw that the cells' power plants (mitochondria) were getting damaged, while the muscle's natural repair program was basically stuck.

**What are the potential implications of this finding for your field of research?**

It highlights a crucial clinical challenge. Simply giving the body back the missing protein through gene therapy might not be enough to fully cure the disease once it has started. If the muscle cells are already choked with damaged mitochondria and a stalled cleanup machinery, we have to find new, secondary drug targets to clear this ‘roadblock’ alongside treating the primary genetic defect.

**Figure:**
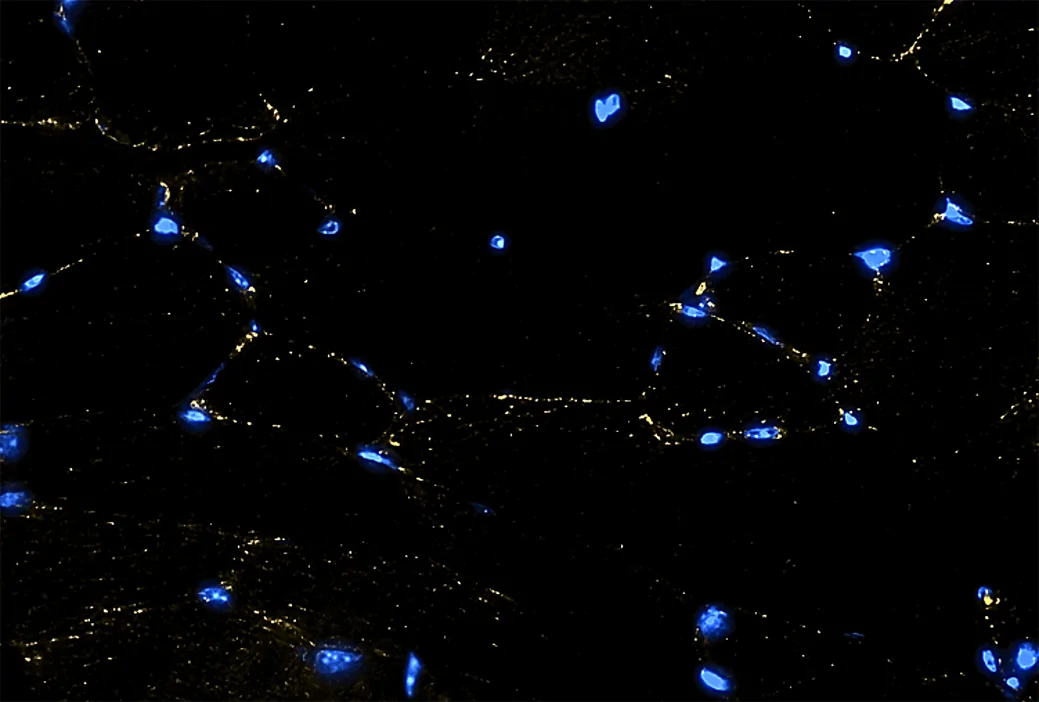
Immunofluorescent image showing the delicate, reticular mitochondrial network (yellow) in healthy mouse skeletal muscle, which becomes severely disrupted in dysferlinopathy.

**Which part of this research project was the most rewarding?**

The most rewarding part was a real ‘detective’ moment with a specific gene called *Prkn*. When our initial investigation showed a drop in this gene, I dug into the literature. I found out that *Prkn* was once described as a key marker for this disease in humans, but it later disappeared from biomarker panels because the signal was too unstable to validate. Our mouse model mirrored this exact historical trajectory. To be honest, we didn't fully solve this mystery in the end. I still strongly believe there is a hidden mechanism there that we are currently missing. But feeling that lingering unknown is exactly how a researcher's obsession is born. Having that unanswered question right in front of you is incredibly inspiring.

**What do you enjoy most about being an early-career researcher?**

In routine clinical diagnostics, you usually just name the disease and move on to the next case. What I enjoy most about research is having the time and freedom to stop and ask ‘why?’. I like being able to combine basic tools, like a microscope, with complex sequencing to figure out how things work. It is a great feeling when you look at a dataset and realise you understand a disease mechanism just a little bit better than you did five minutes ago.

**What piece of advice would you give to the next generation of researchers?**

Remember that statistics and bioinformatics are just tools, but the tissue under the microscope is the biological reality. Today, it is very easy to get lost in big data. If your molecular results go against the classic textbooks, don't automatically assume you made a mistake and throw them away. Sometimes the weird anomaly is not a technical error, but exactly the biological mechanism you are looking for.

**What's next for you?**

We plan to map the genetic and morphological landscape of this mouse model at much more advanced stages of the disease. Ultimately, we want to test a new gene therapy we have developed.
